# Highlights from the 38th SABCS annual meeting, 8th – 12th December 2015, San Antonio, USA

**DOI:** 10.3332/ecancer.2016.618

**Published:** 2016-02-02

**Authors:** Linda Cairns, Giuseppe Curigliano

**Affiliations:** European Institute of Oncology, Via Ripamonti 435, Milan 20141, Italy

**Keywords:** SABCS, breast cancer

## Abstract

The 2015 San Antonio Breast Cancer Symposium (SABCS) annual meeting highlighted the latest discoveries in breast cancer research and as ever provided a unique opportunity for investigators from all over the world to meet and network. With the rapidly increasing pace of discoveries in the basic, translational, and clinical sciences, mainly because of the advent of new technologies, cancer researchers are making rapid progress that is having significant patient benefit. This year’s meeting featured studies on targeted therapy plus endocrine therapy for metastatic disease with a mutation of PIK3CA, chemotherapy combinations for HER-2-positive disease, long-term outcomes of different surgeries for early-stage cancers, and the first-ever trial of a receptor activator of nuclear factor kappa-B ligand (RANKL) inhibitor as an adjuvant treatment for breast cancer in postmenopausal women. In the educational session, there was significant emphasis on the role of metabolic syndrome and lifestyle on breast cancer outcome.

## Metabolic Syndromes

The ‘Metabolic Syndrome and Obesity in Breast Cancer’ educational session moderated by Carlos Arteaga opened with Douglas Yee who discussed the various metabolic pathways involved in breast cancer progression and the potential to target key components of these pathways for treatment. Obesity and insulin resistance are linked. Elevated levels of insulin and insulin like growth factors (IGFs) increase breast cancer risk and poor outcome in patients. This has led to the development of therapies that target the IGF receptor for breast cancer. In contrast to receptor targeting strategies, the inhibition of downstream signaling pathways such as mTORC1 have been successful in HR-positive breast cancer.

In the same session, Dr Mantzoros discussed recent insights into the role of adiponectin in breast cancer especially the potential link between adiponectin and breast carcinoma. Adiponectin, an adipocyte-secreted hormone, is inversely associated with insulin resistance and is considered to be one of the key factors in the connection between obesity and breast cancer.

Pamela Goodwin discussed the potential use of metformin to improve breast cancer outcomes. Obesity is associated with increased risk of breast cancer in premenopausal triple-negative breast cancer (TNBC) and postmenopausal mainly oestrogen receptor positive (ER+). Metformin, commonly used to treat type-2 diabetes, has been associated with lower breast cancer risk and improved outcomes in observational studies. Dr Goodwin hypothesised that the mode of action may be either indirect i.e. by lowering circulating insulin leading to reduced insulin receptor-mediated activation of PI3K and RAS pathways or more directly via an effect on mitochondria leading to mTOR inhibition.

The large international phase Ill trial (NCIC MA.32) was set up to examine the impact of metformin in early breast cancer survivors. Subjects were randomly assigned to metformin or a placebo for five years. Baseline weight and metabolic measurements were taken and the majority of subjects were overweight or obese in both arms. Changes in weight, body mass index (BMI), and blood variables were measured at six monthly intervals. Metformin was found to have a beneficial effect on weight, BMI, and metabolic variables in the study group. They also demonstrated an improvement in several metabolic factors associated with insulin resistance in the patients enrolled in the trial. The beneficial effects were regardless of initial weight or degree of insulin resistance.

## Advanced Breast Cancer

The Management of Advanced Breast Cancer (ABC) educational session opened with Fatima Cardoso discussing the International Consensus Guidelines for the treatment of ABC. The ABC guidelines, developed by ESO and ESMO and endorsed by ASCO are as follows (Annals of Oncology 00: 1–18, 2014 doi:10.1093/annonc/mdu385 - ESO-ESMO 2nd international consensus guidelines for advanced breast cancer (ABC2)† F Cardoso *et al*):

- All ABC patients should be offered comprehensive, culturally sensitive, up-to-date easy to understand information about their disease and its management.
- Specialised oncology nurses (if possible specialised breast nurses) should be part of the multidisciplinary team managing ABC patients.
- Validated instruments should be considered for patients to report the symptoms of disease and side-effects of treatment they experience as a regular part of their clinical care. These patient-reported outcome (PRO) instruments should be simple and user-friendly to facilitate their use in clinical practice. This systematic monitoring will help in the communication between patient and doctor, allow better quality of life (QOL), and may better characterise the toxicities of all anticancer therapies.
- The age of the patient should not be the sole reason to withhold effective therapy (in elderly patients) nor to overtreat (in young patients). Age alone should not determine the type and intensity of treatment.
- Treatment decision should take into account both HR and HER-2 status and also previous therapy.

The European parliament this year made a declaration that all ABC patients should be treated in specialised centres. Dr. Cardoso also emphasised the importance of tumour registries so that the exact number of individuals with ABC can be better calculated worldwide. An update of these guidelines will be presented at the ABC4 meeting in Lisbon in 2017.

Dr. Dickler discussed **‘**Longitudinal strategies in ER+ disease’. Endocrine therapy is a well-tolerated treatment for hormone receptor-positive (HR+) breast cancer and is offered as first-line treatment to patients with no evidence of clinically significant disease. Aromatase inhibitors (AIs), tamoxifen, and fulvestrant all target the oestrogen receptor and provide effective therapy with a durable response. The decision to offer chemotherapy or endocrine therapy should depend on disease burden, biological subtype (e.g. HER-2 status), menopausal status, and prior endocrine therapy.

The sequential use of anti-ER therapy with or without chemotherapy or targeted therapies has been tested in various ongoing trials. Within HR+ breast cancer, there is molecular diversity that can influence responsiveness to endocrine therapy. In addition, recent insight into the complex interactions between the oestrogen receptor and cell cycle survival and/or growth factor signaling pathways have uncovered potential mechanisms of endocrine therapy resistance in HR+ breast cancer leading to new treatment options. These include the cell cycle inhibitor palbociclib and the combination of an mTOR inhibitor, everolimus. These targeted agents appear to increase progression-free survival (PFS) when added to endocrine therapy, although overall survival (OS) benefits are still unclear. Biomarkers will be essential to understand which patients will benefit from which sequence/combination.

In advanced breast cancer, there still remains controversies and variations in practice for many of the chemotherapeutic agents used. In the presentation ‘Tailoring chemotherapy’ Dr Ring stressed the importance of considering all factors when deciding between combinations or sequential single agent chemotherapy. The duration of therapy and the biology of the tumour should be taken into account. He concluded that in general sequential monotherapy is preferred except for patients in which response is required because of aggressive disease. In general longer courses of chemotherapy are superior, however the problem is toxicity. Chemotherapy duration should take into account:

- Patient’s wishes
- Response to initial therapy
- Residual disease
- HER-2 status
- ER status
- Patient tolerance

Chemotherapy choice should depend on the biology of the disease and emerging data will be important for these decisions.

The final presentation of this session ‘Regimens in HER-2+ disease’ was presented by Dr Hurvitz. HER-2-targeted therapy has substantially improved the natural history of HER-2-positive metastatic breast. The current HER-2-targeted therapies, trastuzumab, lapatinib, pertuzumab, and trastuzumab-emtansine (TDM1) have improved the survival rates but in most cases the disease will progress even with these targeted therapies.

The phase III CLEOPATRA study showed a significant 15.7-month increase in median OS over 50 months of follow-up with the addition of pertuzumab to the trastuzumab and docetaxel arm in the first-line treatment of women with HER-2 metastatic breast cancer. There was also a significant improvement in the progression-free survival (PFS) with the pertuzumab-containing regimen. The (Advanced Breast Cancer 3) (ABC3) consensus states trastuzumab with pertuzumab and a taxane is the preferred therapy at the moment.

The phase III EMILIA study evaluated T-DM1 in patients with HER-2-positive metastatic breast cancer who had previously received Herceptin and a taxane-based chemotherapy. This study provided convincing evidence that TDM-1 is efficacious in refractory metastatic breast cancer and provides clinicians with an effective new treatment in this setting. Recent studies have focused on the use of T-DM1 in combination with additional therapies as well as the use of T-DM1 in the adjuvant and neoadjuvant setting (http://www.oncolink.org/conferences/article.cfm?id=6727).

**Figure 1.**
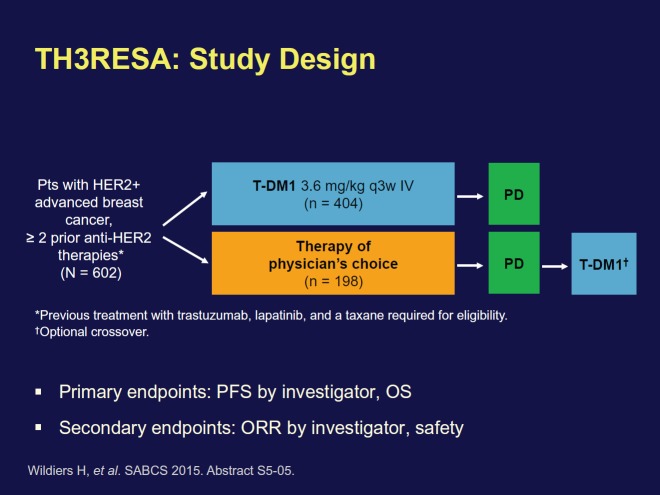
TH3RESA trial design. Reprinted with kind permission from Hans Wildiers.

The results from the phase III TH3RESA trial (Figure 1), which was designed to study TDM1 in a more advanced setting, demonstrated that T-DM1 improved OS for heavily pretreated patients with HER-2-positive breast cancer.

All the patients enrolled in TH3RESA had been previously treated with a chemotherapy regimen that included a taxane and at least two HER-2-targeted agents; trastuzumab and lapatinib. Median survival was significantly longer in patients receiving T-DM1 (22.7 months) compared to patients treated according to the physician’s choice (15.8 months). The benefit was regardless of age, hormone-receptor status, visceral involvement, and number of prior treatment regimens. T-DM1 now represents a good treatment option even for patients who have already received two or more HER-2-targeted treatment regimens and are in progression.

‘Previously results from TH3RESA showed that T-DM1 progression-free survival was nearly doubled’, commented Hans Wildiers at the press conference. ‘The latest results indicate that T-DM1 also increases overall survival in heavily pretreated patients. This is significant as many breast cancer therapies increase progression-free survival but not overall survival’, continued Wildiers.

The first results from the phase III BELLE-2 trial were presented. Activation of the PI3K pathway can promote resistant to endocrine therapies. The concept behind the BELLE-2 was to see if the addition of the PI3K inhibitor (buparlisib) tofulvestrant in patients who no longer respond to AIs could improve outcome. Following 14 days of fulvestrant, patients were randomised to either buparlisib or placebo. In addition blood samples were taken at the start of the trail to analyse for the presence of PIK3CA mutations. The whole study population benefited from the buparlisib–fulvestrant combination; PFS of 6.9 months in the arm buparlisib plus fulvestrant compared to 3.2 in the fulvestrant alone arm. Further patients with the mutant PIK3CA also had better buparlisib plus fulvestrant when compared with those who received fulvestrant but only slightly better (seven months) than the whole population. ‘This is the first time that inhibiting the PI3K pathway may be a viable option for patients with endocrine therapy-resistant breast cancer,’ said José Baselga, the lead researcher on this study.

Mutations in the oestrogen receptor are more common in patients with advanced, ER+ breast cancer and they result in the cells becoming independent of oestrogen for growth and no longer respond to tamoxifen and AIs. The group of Chandarlapaty wanted to ask the question ‘Are mutations in the oestrogen receptor common in patients with advanced breast cancer’? And do they have an effect on outcomes? By evaluating blood samples from 541 of the 724 patients enrolled in BOLERO-2 they detected the D538G ESR1 mutation in samples from 83 patients, the Y537S ESR1 mutation in samples from 42 patients, and both mutations in samples from 30 patients. Those patients who had a D538G and/or a Y537S mutation had significantly worse median OS. It is not clear if the AI could be responsible for these mutations. Further, the data from the Bolero trial indicated that wild-type and patients with D538G mutation derived a benefit from the addition of everolimus, whereas those with Y537S mutation did not. These results require further validation. The potential to use these mutations as potential markers for healthy women who may be at risk of developing breast cancer is still an open question.

## Early Breast Cancer

**Figure 2.**
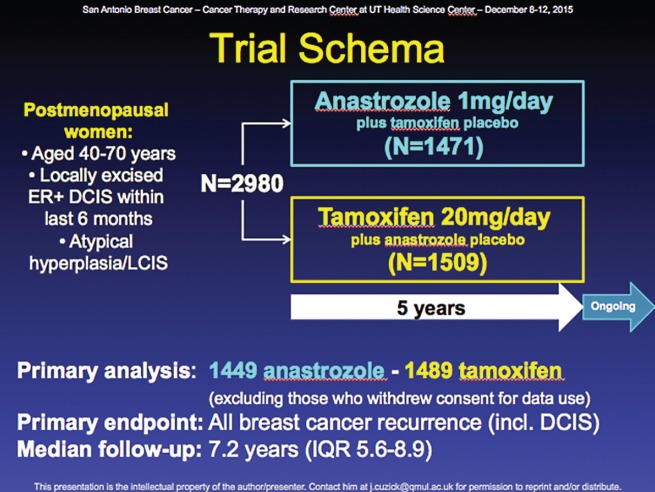
ABCSG-18 trial design.

The first trial examining adjuvant denosumab (Xgeva, Amgen) as a treatment for breast cancer was presented by Michael Gnant, MD, from the Medical University of Vienna in Austria. The phase 3 ABCSG-18 clinical trial (Figure 2) compares denosumab with placebo in combination with adjuvant AI therapy in 3000-plus postmenopausal women with early-stage hormone-receptor-positive breast cancer.

**Figure 3.**
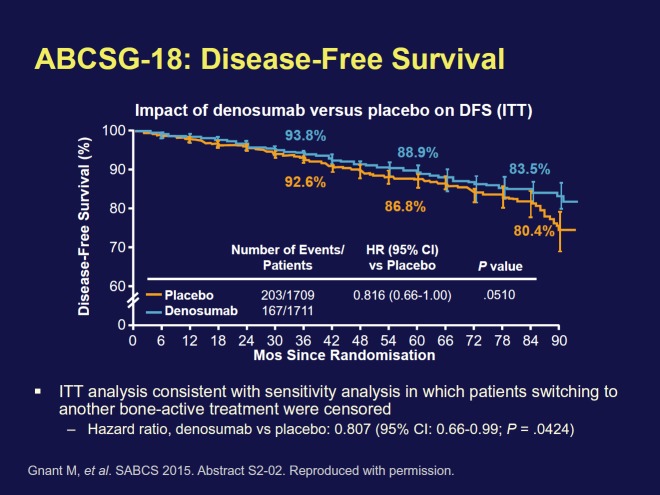
Disease free survival in the ABCSG-18 trial.

Denosumab is approved as a treatment to increase bone mass in breast cancer patients receiving endocrine therapy who are at high risk for fracture. Adjuvant denosumab increased DFS in AI-treated postmenopausal women with early HR+ breast cancer [Hazard ratio (ITT): 0.82 (95% CI (confidence interval): 0.66–1.00; P = .0515); Hazard ratio (sensitivity analysis): 0.81 (95% CI: 0.66–0.99; P = .0424)]. Denosumab was safely administered (Figure 3). No differences in adverse events (AEs), serious AEs with denosumab versus placebo was seen. No patient experienced necrosis of the jaw, or atypical fracture. Based on increased disease-free survival (DFS), safety profile, and reduction in fractures, the authors conclude that denosumab should be made available to postmenopausal patients with HR+ breast cancer on AIs.

Dr Gnant explained that there is already evidence that bisphosphonates, another type of bone-building agent, are an effective adjuvant treatment. They produce a reduction in the recurrence rate of about 30% in postmenopausal women with early-stage disease. If the data on denosumab, which is the first-in-class RANKL inhibitor, are positive, ‘it’s going to be hard to deny use of one of the two strategies in patients’, he said. ‘So what’s the mechanism of action’? Dr Gnant speculated that ‘Silencing the bone marrow micro-environment may reduce the chance of dormant cancer cells of waking up’.

Patients with residual disease after neoadjuvant chemotherapy are potentially chemoresistant, but there have been no large-scale clinical trials to test whether adjuvant systemic chemotherapy could benefit these patients. Data from the Create-X trial, presented by Dr Toi, found that capecitabine improved outcomes for breast cancer patients with residual disease after neoadjuvant chemotherapy. After two years of follow-up, DFS was significantly improved by the addition of capecitabine in the adjuvant setting. OS was improved in patients with triple negative residual and node positive disease following neoadjuvant chemotherapy. Patients who were assigned capecitabine had a 30% reduced risk of disease recurrence compared with those assigned no capecitabine.

**Figure 4.**
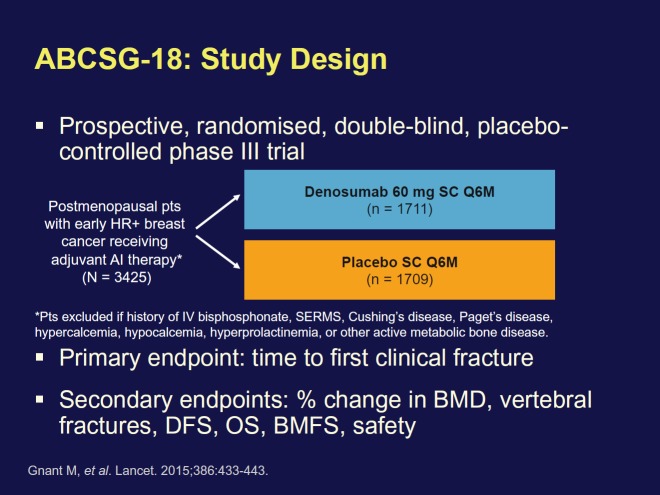
IBIS-11 trial design.

In the chemopreventive setting, Dr Cuzick presented the ten year follow-up of the IBIS-II DCIS clinical trial (Figure 4), on behalf of the IBIS working party. This study, which followed on from the ATAC trial (Arimidex, tamoxifen, alone or combination) looked at disease recurrence in postmenopausal women with ductal carcinoma *in situ* (DCIS) treated with either tamoxifen or the AI anastrozole. In this multicentre, randomised, placebo-controlled trial, 2980 postmenopausal women with locally excised hormone receptor-positive DCIS were enrolled; 1471 were randomly assigned 1mg/day anastrozole, and 1509 were randomly assigned 20 mg/day tamoxifen.

### Reprinted with kind permission from Jack Cuzick

In this trial, it was found that the women in both the anastrozole and tamoxifen groups had similar overall efficacy, with slightly better outcomes for those who took anastrozole. However, the side-effects were dramatically different. More strokes and fractures were seen in the anastrozole arm, but these women tended to have less endometrial, ovarian, and skin cancers compared with those who took tamoxifen; tamoxifen is known to cause gynaecological cancers. However, among those receiving tamoxifen had more thromboembolic events and gynaecological complications.

So in summary postmenopausal women with DCIS had similar outcomes with tamoxifen or the AI anastrozole. The clinician’s choice should take into account the patient background and comorbidities when deciding between the two options.

Following on from this, Dr Patricia Ganz presented the results of a very similar trial (NSABP B-35 trial) but she focused on PRO. This trial compared anastrazole to tamoxifen in postmenopausal women with DCIS.

Just over 3000 postmenopausal women were enrolled and the primary endpoint indicated that anastrozole was slightly but significantly better than tamoxifen in terms of breast cancer-free interval, and was more beneficial in younger women.

The quality of life (QOL) study, which was embedded in this study (1193 patients), looking at symptoms such as hot flashes, vaginal dryness, muscle and joint aches indicated no differences in QOL outcomes.

The results from these two studies were published simultaneously in *Lancet* (December 10th).

There is mounting evidence that breast conserving therapy (BCT) is as good as if not better than mastectomy for patients with early breast cancer. Data was collected from a study which compared the ten-year OS and DFS after BCT plus radiation therapy with mastectomy (without radiation therapy) in women with early stage breast cancer. By analysing data from the Netherlands Cancer Registry (37,207 women with early breast cancer between 2000 and 2004 to estimate ten-year OS, and 7552 patients with similar characteristics diagnosed in 2003 to estimate ten-year DFS). Dr Siesling showed that patients who received BCT had a ten-year OS of 76.8% compared to 59.7% for mastectomy. The ten-year DFS was 83.6% versus 81.5% for the two groups respectively. ‘We think that radiation therapy may have played an important role in the difference in the outcomes from both treatments, although we cannot prove it with our data’, Siesling said. ‘We suggest that BCT should be the treatment of choice, especially in T1N0 stage breast cancer when it is medically feasible and according to the patient’s wish’, she added. These results will be important in the shared decision-making process and improve the quality of breast cancer care.

In the early breast cancer setting, the Multidisciplinary Approach to Novel Therapies in Cardiology Oncology Research (MANTICORE) trial should be also mentioned. This is a study for patients with HER-2-positve early-stage breast cancer. It is the first randomised controlled study to evaluate the use of antihypertensive agents for the prevention of cardiac damage associated with trastuzumab treatment. The MANTICORE researchers assessed whether one year of treatment with the ACE inhibitor perindopril or the beta-blocker bisoprolol can prevent the left ventricular remodelling (measured with MRI) associated with one year of trastuzumab therapy.

In this trial, 99 patients received either the drug perindopril, or the drug bisoprolol, or a placebo. Bisoprolol belongs to a class of drugs called beta-blockers. Perindopril is a type of drug known as a angiotensin-converting enzyme (ACE) inhibitor.

The study found that women taking either perindopril or bisoprolol had fewer signs of heart weakening than the women in the placebo group. Heart protection effects were also found in the group of women taking perindopril. (Reference: Pituskin E, Mackey JR, Koshman S, *et al*). Prophylactic beta blockade preserves left ventricular ejection fraction in HER-2-overexpresssing breast cancer patients receiving trastuzumab: Primary results of the MANTICORE randomised controlled trial. Presented at the San Antonio Breast Cancer Symposium; 9–12 December 2015; San Antonio, TX).

Samuel Aparicio gave the plenary lecture entitled Clonal Dynamics and Breast Cancer subtypes**.** Breast cancers exhibit interpatient and intratumoural genomic variability which underpins our understanding of intrinsic drivers of the disease. Patterns of genomic heterogeneity will be important for treatment decisions and may also prove to be prognostic. Next generation sequencing has redefined the landscape of primary breast cancer subtypes into many molecularly defined subgroups and now the identification of driver mutations in primary cancers will be important. To date at least ten primary breast cancer subtypes exist. In breast cancer 40 mutational drivers have been identified. These mutations segregate by tumour histological subtype i.e. ER+, TNBC, etc.

Apart from interpatient breast cancer heterogeneity it is accepted that most tumours are made up of dynamic clones that evolve with the progression of the disease. The evolution of the clonal composition has particular significance for cancer medicine. Over the last five years next generation sequencing of tumours and methods for single cell analysis have opened up this approach for solid epithelial malignancies.

Dr Samuel Aparicio posed the question ‘How much clonal variation is there in TNBC’. His team found a wide variation in clonal common myeloid progenitor (CMP) between TNBC of the same stage. One important factor that influences the clonal complexity of the tumour was derived from engraftment experiments. Engraftments were influenced by the site of transplant, indicating that the stroma influences the propagation of the engrafted clone. So in summary clonal dyanamics of tumours can be measured in model systems. Primary breast cancers exhibit a wide variety of clonal structure at diagnosis. Some aspects of clonal complexity can be captured in the model systems.

The presence of tumour infiltrating lymphocytes (TILs) in invasive breast carcinoma especially in the TNBC and HER-2+ subtypes is associated with a better prognosis. So far, TILs have not been investigated in invasive lobular breast cancer (ILBC). The group of Dr Desmedt therefore decided to assess the distribution of stromal TILs in ILBC and to try to correlate their presence as pathological markers. They also want to investigate if there is a link with recurrent genomic alterations.

By analysing over 600 ILBC tumours, they found that in general most tumours have low numbers of TILs. However they found that higher numbers of TILs were significantly associated with ER-, high proliferative tumours, younger age at diagnosis, and axillary lymph node involvement.

Also as they had the genomic data on these tumours, they could look at the mutations. With this they found that greater numbers of TILs were observed in tumours harbouring ARID1A, BRCA2, KMT2C, and TP53 mutations, as well as chr3p21.31 and chr8q24.23 (PTK2) loss. Surprisingly, tumours with greater numbers of TILs were associated with worst prognosis variables at the univariate analysis.

It is now evident that genetic intratumour heterogeneity exists in many tumours. This heterogeneity may arise because of selective pressures, the most important of these being chemotherapy. This year Anne-LiseBørresen-Dalegave in the 8th AACR Distinguished Lectureship, said her laboratory uses a systems biology approach to study breast cancer using high-dimensional data in integrated approaches. She discussed data indicating that intratumour heterogeneity in breast cancer arises in response to neoadjuvant therapy. This heterogeneity probably plays an important role in developing resistance to chemotherapy and stratification is important to better understand breast cancer.

Data from the phase III DBCG77B clinical trial, presented this year demonstrated that premenopausal women with the luminal A subtype of breast cancer had comparable ten-year DFS rates regardless of whether or not they received adjuvant chemotherapy. Luminal A is a relatively common subtype of breast cancer, with high expression of the oestrogen (ER) and progesterone receptors (PR), and low Ki67 index and low HER-2. Between 1977 and 1983, 1146 premenopausal women who had lymph node-positive invasive breast cancer that was larger than 5 cm were randomised to two chemotherapy arms and two no-chemotherapy arms. After analysing tissue samples for the presence of ER, PR, HER-2, and Ki67, Dr Nielsen and colleagues identified 165 which were luminal A subtype. Within this group there was no difference in ten-year invasive DFS rates between women with luminal A disease who did and did not receive chemotherapy.

The importance of residual disease after neoadjuvant chemotherapy was a topic of various presentations this year at SABCS. Results from the randomised phase II CALGB/Alliance 40603 clinical trial indicated that patients with cancer TNBC who had a pathologic complete response (pCR) after neoadjuvant chemotherapy had a better OS compared with those who had residual invasive disease at surgery.

Circulating tumour cells (CTCs) are of prognostic relevance in early as well as metastatic breast cancer. Persisting CTCs immediately after chemotherapy are known to indicate poor prognosis but there is lack of data on the prognostic role of CTCs in long-term follow-up. Hence the prognostic value of CTCs two years after chemotherapy was analysed. Results from the adjuvant SUCCESS A trial, presented by Dr Janni, indicated that persistence of circulating tumour cells, two years after adjuvant therapy was associated with poor prognosis. Patients with persistent CTCs during long-term follow-up may serve as surveillance marker and should receive further therapy.

Several recent studies have implicated increased levels of the enzyme APOBEC3B in oestrogen receptor–positive breast cancer with poor patient outcomes. Dr Rueben Harris wanted to understand if APOBEC3B actually drives these poor outcomes. To do this in pre-clinical studies they transplanted mice with either cell lines expressing high levels of APOBEC3B or cells in which the levels of APOBEC3B had been reduced by a shRNA. After treating the mice with tamoxifen the mice with high levels of APOBEC3B developed tamoxifen resistance significantly more rapidly than those with the reduced levels. In collaboration with the group of Drs Martens and Span in the Netherlands, they analysed APOBEC3B levels in tumours samples from patients with recurrent ER+ breast cancer. They found that patients with high levels of APOBEC3B progressed significantly sooner after starting tamoxifen therapy than those with low levels of APOBEC3B. So in summary, increased levels of APOBEC3B can significantly reduce tamoxifen responses, suggesting that APOBEC3B drives resistance to tamoxifen. Harris explained that ‘APOBEC3B is an enzyme that causes genetic mutations’ suggesting that it is these mutations that probably drive tamoxifen resistance.

Cyclin-dependent kianses (CDKs) have been known to be critical regulators of cell cycle progression and mutations in these genes have been implicated in cancer. In breast cancer, they have been associated with various molecular subtypes, prognosis, and response to therapy. The concept of blocking CDKs has been around for some time but they have only recently seen clinical success. The first generation CDCK inhibitors were not targeted molecules but pan inhibitors with significant toxicity. Now more specific molecular targeted agents exist. However it is important to identify which patient populations are most likely to benefit.

Palbociclib (PD-0332991, Pfizer) is a first-in-class CDK 4/6 specific inhibitor. Dr Finn explained that pre-clinical studies identified that ER+ breast cancer models were most sensitive to growth inhibition with palbociclib and identified a synergistic interaction in inhibiting proliferation in combination with tamoxifen [1]. Sensitive ER+ breast cancer cell lines treated with the combination went into senescence. These data served as a hypothesis for the phase II Paloma-1/ TRIO 18 study of palbociclib +letrozole versus letrozole alone [2]. A significant improvement in PFS was observed with the combination and resulted in the approval of palbociclib plus letrozole as first line therapy for advanced postmenopausal ER+ breast cancer. In more advanced settings a benefit was seen in combination with fulvestrant [3]. Currently there are several other CDK 4/6 inhibitors in development in breast cancer and other malignancies including abemaciclib (LY2835219, EliLilly) and ribociclib (LEE011, Novartis).

## Immunotherapy in Breast Cancer

At this years SABCS meeting, there were a significant number of presentations and posters on this subject indicating that this is a hotly emerging line of research.

Tumour-infiltrating lymphocytes (TILs) are recruited into tumours in an attempt to control its growth and there is evidence that there is a positive association between the amount of TILs present at diagnosis and outcome in various cancers. A pooled analysis of studies that investigated the presence of TILs in TNBC patients treated with chemotherapy was presented by Dr Loi. Data from 991 TNBC patients included in six randomised clinical trials indicated a strong prognostic role of TILs in TNBC. He summarised that patients with TNBC are potential candidates for immunotherapy clinical trials.

Immunotherapy using antibodies targeting the cytotoxic T-lymphotcytes associated protein 4 (CTLA-4) or PD-1 pathways have demonstrated a clear clinical efficacy in a few cancers. The PD-1 receptor and its ligand PD-L1 are key therapeutic targets in the reactivation of the immune response against cancer**.** Avelumab an anti-PD-L1 antibody was tested in a cohort of patients with locally advanced or metastatic breast cancer refractory to or progressing after standard-of-care therapy. The main investigator, Dr Dirix, explained that despite an acceptable safety profile the agent demonstrated only modest activity in these patients. In a subgroup of patients, those with TNBC, the presence of PD-L1 expressing immune cells may be a sign of a better clinical response to Avelumab. Immunotherapy is a promising tool for breast cancer clinicians and over the next few years there will be a surge in the amount of these trials.

## Precision Medicine in Breast Cancer

A mini symposium addressing the use of genome sequencing to identify driver mutations in breast cancer was held on the final day of this year’s conference. Between 10–20 molecular alterations are being investigated in the context of biomarker-driven therapeutic trials, including PIK3CA, AKT1, ERBB2, PTEN, BRCA1/2, ESR1 mutations. However it is important to identify which of the mutations are actually drivers. As noted earlier, it appears that there are mutations in the PIK3CA gene that are associated with lower sensitivity HER-2 inhibition in the neoadjuvant setting. The second challenge will be to generate genomic tests that predict the sensitivity of therapies that target pathways, like mTOR or CDK4 inhibitors. In this context gene expression will be useful in quantifying pathway activation. The third challenge will be to predict much earlier resistance to targeted therapies. The development of liquid biopsies (circulating DNA)will become more and more important for early diagnosis of resistance. In the future, it will be valuable to biopsy also the solid tissue as protein identification will become important. Finally, one of the major challenges will be to integrate immune-therapeutics into precision medicine.
